# The relationship between sleep problems and cortisol in people with type 2 diabetes

**DOI:** 10.1016/j.psyneuen.2020.104688

**Published:** 2020-07

**Authors:** Ruth A. Hackett, Zeynep Dal, Andrew Steptoe

**Affiliations:** a Health Psychology Section, Institute of Psychology, Psychiatry and Neuroscience, King's College London, Great Maze Pond, London, SE1 9RT; b Department of Behavioural Science and Health, University College London, London, UK

**Keywords:** Cortisol, sleep problems, stress response, Type 2 diabetes

## Abstract

**Background:**

Sleep problems are linked with negative health outcomes, including coronary heart disease. Neuroendocrine dysfunction has been associated with sleep problems and may be a pathway linking sleep and ill health. Dysregulated cortisol output has observed in people with type 2 diabetes (T2D), though little is known about the links between sleep and cortisol in this population at high risk of coronary disease.

**Method:**

This study investigated the association between sleep problems and cortisol over the course of an ordinary day and in response to acute laboratory stress in a sample of 129 individuals with T2D. Sleep problems were assessed using the Jenkins sleep problems questionnaire. Mental stress was induced using two five-minute laboratory stress tasks: a mirror-tracing task and the Stroop color-word interference task.

**Results:**

Sleep problems were positively associated with daily cortisol area under the curve (*B* = 17.051, *C.I.* = 6.547 to 27.554, *p* = 0.002) adjusting for age, sex, marital status, education, household income, body mass index and smoking; suggesting that those with greater sleep problems had greater cortisol concentrations over the course of an ordinary day. Participants reporting greater sleep problems also had raised evening cortisol levels (*B* = 0.96, *C.I.* = 0.176 to 1.746, *p* = 0.017) in adjusted models. In the laboratory sleep problems were negatively associated with cortisol immediately post-task (*B* = -0.030, *C.I.* = -0.059 to 0.000, *p* = 0.048) and 45 minutes post-task (*B* = -0.037, *C.I.* = -0.072 to -0.002, *p* = 0.039) in fully adjusted models; indicating that those who experienced greater sleep problems had lower cortisol concentrations after stress.

**Conclusions:**

Sleep problems were associated with disturbances in cortisol responses to stress, as well as changes diurnal cortisol output in people with T2D. Further research is needed to assess if neuroendocrine disturbance increases the risk of cardiovascular disease in this population.

## Highlights

• Sleep problems are associated with ill-health and neuroendocrine dysfunction
• Cortisol output is altered in type 2 diabetes.
• Sleep problems are linked with raised daily cortisol levels in people with diabetes.
• Sleep problems are linked with lower cortisol stress responses in type 2 diabetes.
• Altered cortisol may be a pathway linking sleep and ill-health in type 2 diabetes.

## 1 Introduction

Sleep is a vital process that is essential for survival. Sleep quality is a key component of sleep health (Buysse, 2014) which includes the subjective assessment of the time taken to fall asleep, the frequency of disturbed sleep and feeling rested on waking, is important for optimal daytime functioning and wellbeing (Chrousos et al., 2016). However, complaints about sleep quality are common. Results from international (Léger et al., 2008) and British surveys (Calem et al., 2012) suggest that over a third of the adult population experience poor quality sleep on a regular basis.

Poor sleep quality has been linked with negative health outcomes. Adults who report sleep problems are more likely to have hypertension, obesity and coronary heart disease (CHD) than their counterparts without sleep problems (Koyanagi et al., 2014). Meta-analytic evidence has detected a relationship between short sleep duration and incident hypertension (Itani et al., 2017). However, studies investigating the prospective link between poor sleep quality and hypertension have produced mixed results (St-Onge et al., 2016). Pooled evidence indicates that short sleep duration is a risk factor for obesity (Itani et al., 2017). While poor sleep quality has been linked with the development of the metabolic syndrome in middle-aged and older adults (Troxel et al., 2010).

There is considerable evidence linking sleep with cardiovascular disease (CVD). Short sleep duration has been linked with the development of CHD and CVD, as confirmed in several meta-analytic studies (Cappuccio et al., 2011; Itani et al., 2017). There is also evidence that insomnia (defined as difficulty initiating/maintaining sleep or the presence of disturbed/restless sleep) is a risk factor later CVD (Sofi et al., 2014). Furthermore, short sleep duration (Cappuccio et al., 2011; Itani et al., 2017) and sleep-breathing disorders (St-Onge et al., 2016) have been associated with CVD and all-cause mortality.

Although the findings linking sleep and poor health demonstrate its clinical importance, the mechanisms underlying the association remain unclear. One potential pathway that could be involved is neuroendocrine dysfunction, as sleep has a modulatory effect on hypothalamic pituitary adrenal (HPA) axis functioning (Chrousos et al., 2016). Optimal sleep is associated with a healthy diurnal profile of cortisol release (Chrousos et al., 2016), characterized by raised cortisol concentrations in morning on waking, followed by a decline over the course of the day before sleep (Adam and Kumari, 2009).

However, findings concerning the link between sleep problems and basal cortisol release are mixed and are complicated by the use of varied sleep deprivation methodologies, discrepancies between objective and self-reported sleep parameters (Chrousos et al., 2016), as well as the type (e.g. urinary, salivary) and frequency of cortisol sample collection (Adam and Kumari, 2009).

Observational studies that have assessed diurnal salivary cortisol output in relation to sleep quality are equivocal. Some studies have reported a link between poor sleep quality and low cortisol output in the morning (Backhaus et al., 2004; Castro-Diehl et al., 2015; Hansen et al., 2012), while others have found associations with high morning concentrations (Abell et al., 2016) or no association at all (Kumari et al., 2009; Van Lenten and Doane, 2016; Zhang et al., 2011). A flatter slope in the decline in cortisol across the day has been linked with sleep problems in several (Castro-Diehl et al., 2015; Huang et al., 2017; Kumari et al., 2009) but not all studies (Abell et al., 2016; Hansen et al., 2012; Van Lenten and Doane, 2016). While both raised (Kumari et al., 2009) and low evening cortisol levels (Hansen et al., 2012) have been associated with sleep problems. In terms of overall diurnal output some (Hansen et al., 2012), but not all studies (Castro-Diehl et al., 2015) have observed a link between raised cortisol output and disturbed sleep.

There is also evidence that poor sleep may not only impact the basal activity of the HPA axis but also the reactivity of this system to stress (van Dalfsen and Markus, 2018). However, findings in this area have also been varied. Some studies have linked poor sleep quality with greater cortisol reactively to laboratory stress (Bassett et al., 2015; Goodin et al., 2012), while others have observed a blunted response to stress (Wright et al., 2007) or no association at all (Brooks and Robles, 2009).

Despite this evidence, most of the research to date has been conducted with healthy samples, but sleep problems might pose an additional risk for people with type 2 diabetes (T2D). Meta-analytic evidence indicates that poor sleep quality is a risk factor for T2D (Cappuccio et al., 2010). There is a recognized association between sleep problems and CVD (Sofi et al., 2014) and CVD is the leading cause of death in people with diabetes (Emerging Risk Factors Collaboration, 2011).

Furthermore, T2D has been linked with neuroendocrine dysfunction. People with T2D have an altered diurnal cortisol rhythm compared to those without T2D (Hackett et al., 2014; Steptoe et al., 2014) and raised evening cortisol concentrations are predictive of new onset T2D in initially healthy samples (Hackett et al., 2016). People with T2D have also been found to have altered cortisol reactivity in laboratory settings, with blunted cortisol levels observed in those with T2D in comparison with controls (Steptoe et al., 2014).

To address the gaps in the literature, the current study investigated the link between sleep problems and cortisol over the day and in response to laboratory stress in people with T2D. We expected that sleep problems would be associated with raised evening cortisol concentrations in a naturalistic setting and with blunted cortisol responses in the laboratory environment.

## 2 Material and methods

### 2.1 Participants

The current study uses data from 129 individuals (81 men and 48 women) diagnosed with T2D who first took part in a laboratory stress trial and then provided cortisol samples over the course of an ordinary day (Steptoe et al., 2014). Participants were recruited from diabetes outpatient and primary clinics in London. Those who were previously diagnosed with any CHD, inflammatory disease or allergies were not able to take part in the study. Those who were diagnosed with a mood disorder were also not included in the study. None of the participants reported having a sleep or an endocrine disorder. Participants were notified not to use any anti-inflammatory/antihistamine medications one week beforehand and were also instructed not to consume any caffeinated beverages and not to smoke 2 hours prior; and to avoid vigorous exercise and alcohol one night prior to the study. Participants who reported having a cold or other infection on day of testing, were given an appointment at another time. All participants provided full written informed consent, and ethical approval was given by National Research Ethics Service.

### 2.2 Sleep problems measures

Sleep problems were assessed using an adapted 5 item version of the Jenkins sleep problems questionnaire (Jenkins et al., 1988). The questionnaire is a measure of perceived sleep quality and assesses sleep issues in the past month with items such as “how often did you have trouble falling asleep” and “how often did you have trouble staying asleep”. In addition to the original 4 items, a fifth item was included “how often in the past month did you have disturbed or restless sleep?” (Kumari et al., 2009). The response options are rated on scale from 1 = ‘not at all’ to 6 = ‘22-31 days’. Scores were averaged with higher scores indicating greater sleep problems. This scale has been previously used in clinical samples (Jenkins et al., 1988) as well as in large epidemiological cohorts (Kumari et al., 2009). The internal consistency (Cronbach α) of the scale was 0.86 in this sample. Subjective stress was measured during the laboratory study using a 7-point scale, with higher scores indicating greater subjective stress.

### 2.3 Other measures

Sociodemographic measures were obtained from participants via self-report questionnaire. Age was recorded in years. Sex (male/female), ethnicity (white/other) and smoking status (yes/no) were coded as binary variables. Marital status was coded into three groups: 0 for married, 1 for single, and 2 for divorced, separated or widowed. Education was assessed in four groups: 1 = no qualifications, 2 = up to O levels (junior high school certificate), 3 = A/ONC levels (high school certificate), 4 = University degree and above. Household income was measured in pounds sterling and grouped into three categories: 0 for less than £20,000, 1 for £20-40,000 and 2 for greater than £40,000. Participants self-reported their diabetes medication (oral anti-diabetics/insulin).

### 2.4 Cortisol sampling over a normal day

Salivary cortisol over the course of a normal day was collected at five time points; immediately after waking, 30 minutes after waking, at 10.00-10.30, 16.00-16.30 and at 20.00-20.30. Participants were instructed not to eat, drink caffeinated beverages or smoke in the 30 minutes before saliva collection. The time of sample collection and violations of the collection procedure were noted by the participant. The cortisol awakening response (CAR) was computed by subtracting the waking cortisol value from the measure taken 30 minutes after waking. The cortisol slope was calculated by regressing cortisol values at time 1, time 3, time 4 and time 5 on time after awakening. This method has previously been described and does not include time 2 in the calculation as the CAR and slope are suggested to be under different neurobiological control systems (Adam and Kumari, 2009).

### 2.5 Cortisol sampling in the laboratory

The testing was completed either in the morning or afternoon in a light- and temperature-controlled laboratory. The time of testing was coded as ‘1 = am’ and ‘2 = pm’. At the start of the session the participants’ height (meters) and weight (kilograms) were measured and this information was used to compute their body mass index (BMI; kilograms/metres^2^). Cardiovascular measures and blood samples were taken as part of the larger trial (Steptoe et al., 2014). These were not assessed in the current study so are not discussed here. Saliva samples for assessment of cortisol were obtained throughout the study using Salivettes (Sarstedt, Leicester, UK). At the start of the study the participant rested for 30 minutes. The baseline sample was taken from the participant at the end of the resting period, along with a subjective stress rating. Next, participants completed the two 5-minute stress-inducing tasks. A saliva sample and stress rating were collected directly afterwards. Additional saliva samples and subjective stress ratings were collected at 20, 45, and 75 min after the tasks were completed.

### 2.6 Mental stress testing

To induce mental stress, participants were given two five-minute tasks: a mirror tracing task and a Stroop color-word interference task. These tasks were administered in random order. For the mirror tracing task, the participant was required to trace a star on a metal plate with a pen but could only see their hand and the star in mirror image. Each time the pen went outside of the borders of the star, the device made a sound indicating a mistake (Lafayette Instruments Corp, Lafayette, IN). Participants were told that an average person could trace the star 5 times in 5 minutes with minimal mistakes. The other stress-inducing task was the Stroop color-word interference task. In this task, participants dealt with target words indicating colors (e.g. red, blue etc.) printed in a different colored ink than the word itself indicated. There were four possible response options (names of colors) written in discordant colors at the bottom of the screen. The task was to choose the word that corresponded to the ink of the target word. These tasks have been used previously in our laboratory (Hackett et al., 2012) and induce comparable stress and task engagement ratings in participants from different socioeconomic groups (Steptoe et al., 2002).

### 2.7 Cortisol assays

The saliva samples were kept at –20 degrees before analysis. Cortisol levels were measured by a time-resolved immunoassay with fluorescence detection at University of Dresden. Intra- and inter assay coefficient of variations (CV) were less than 8%.

### 2.8 Statistical analysis

The laboratory cortisol data was skewed, so it was log transformed prior to the analyses. For the laboratory cortisol data, raw values over the stress testing session and cortisol area under the curve (AUC) with respect to baseline were used for the analysis. The AUC was calculated according to the Pruessner et al. (2003) procedure. For cortisol over the day, raw values, the CAR, the slope and AUC were used. Participants’ cortisol levels over the course an ordinary day and during the laboratory session were evaluated using repeated measures analysis of variance (ANOVA), with time as the within subjects factor. Associations between sleep problems and participant characteristics were assessed using univariate ANOVA for categorical variables and Pearson’s correlations for continuous variables. Multiple regression analysis was used to assess the relationship between sleep problems (as the predictor variable) and cortisol over the day values and laboratory cortisol measures in separate models. Age, sex, marital status, education, household income, BMI and smoking were controlled for in all models. These variables were chosen as covariates because previous studies suggest that they may play a role in cortisol responses (Jones et al., 2012; Kudielka et al., 2004; Roy et al., 1994; Steptoe et al., 2002). In preliminary analyses there were no significant interactions between scores on the sleep problem questionnaire and sex, subjective stress during the laboratory session, time of testing or medication usage on cortisol measures in the study. Therefore, these variables were not included as covariates in the final models. Results are presented as unstandardized regression coefficients (B) with 95% confidence intervals (CI). For illustrative purposes, the participants were divided into two groups using a median split on the average values of all questions from the questionnaire (low vs high sleep problems). Repeated measures ANOVA with cortisol over the day or cortisol over the laboratory session as the within subjects factor and sleep problem group as the between subjects factor was conducted to create these plots. Untransformed values are presented in the text and tables to aid interpretation. All analyses were carried out using SPSS version 24 (SPSS, Chicago, IL).

### 2.9 Sensitivity analysis

In our sample 27 (20.9%) of the participants were non-white. There is evidence to suggest that cortisol output may differ depending on ethnicity (Hajat et al., 2010). Therefore, we assessed whether ethnicity was correlated with any of the cortisol measures and whether there was an interaction between sleep problems and ethnicity on the cortisol values. As a sensitivity check, we also assessed whether the pattern of responses changed when excluding non-white participants from the analyses.

## 3 Results

### 3.1 Participant Characteristics

The characteristics of 129 participants who took part in the study are displayed in Table 1. The mean age was 63.85 (6.87) years with a range of 50-75 years. Most of the participants were male (62.8%) and of white ethnicity (79.1%). The majority were married (51.9%) and educated to degree level (64.3%). Most of the participants were non-smokers (86%), with an average BMI in the obese range 30.64 (5.71) kg/m^2.^ The majority of participants had a low household income of less than £20,000 annually (42.6%). The average sleep problem score was 2.85 (1.34) with a range of 1.00-6.00 Sleep problem scores were not significantly related to age, ethnicity, marital status, education, household income, smoking, BMI, glycated hemoglobin (HbA1c) or subjective stress during the tasks (*p’s* > 0.057). Sleep problem scores were significantly related to sex (*p* = 0.011), with women reporting greater sleep problems on average (3.24 ± 1.40) than men (2.62 ± 1.26). Those taking diabetic medication (oral medication or insulin) were more likely to report sleep problems (*p* = 0.010), than those not on medication (2.99 ± 1.38 and 2.14 ± 0.91, respectively).

**Table 1:** Participant characteristics (n = 129).

Variable	Overall sample	Low sleep problems (*n* = 65)	High sleep problems (*n* = 64)	*p*
Age (years)	63.85 (6.87)	64.26 (7.20)	63.44 (6.54)	0.498
Sex (% men)	81 (62.8%)	48 (59.3%)	33 (40.7%)	0.009
Ethnicity (% white)	102 (79.1%)	49 (48.0%)	53 (52.0%)	0.300
Marital status (% yes)				0.244
Single	28 (21.7%)	16 (24.6%)	12 (18.8%)	
Married	67 (51.9%)	36 (55.4%)	31 (48.4%)	
Divorced or Widowed	34 (26.4%)	13 (20.0%)	21 (32.8%)	
Education (%)				0.470
No qualifications	11 (8.5%)	7 (10.8%)	4 (6.3%)	
O levels (Junior high)	22 (17.1%)	9 (13.8%)	13 (20.3%)	
A/ONC level (High school)	13 (10.1%)	5 (7.7%)	8 (12.5%)	
Degree and above	83 (64.3%)	44 (67.7%)	39 (60.9%)	
Smoking (% yes)	18 (14%)	9 (13.8%)	9 (14.1%)	0.972
BMI (kg/m^2^)	30.64 (5.71)	29.92 (5.07)	31.38 (6.24)	0.147
Household income (%)				0.563
< £20,000	55 (42.6%)	25 (38.5%)	30 (46.9%)	
£20-40,000	37 (28.7%)	19 (29.2%)	18 (28.1%)	
> £40,000	37 (28.7%)	21 (32.3%)	16 (25%)	
HbA1c (%)*	7.30 (1.46)	7.30 (1.57)	7.30 (1.34)	0.976
Diabetic medication (% yes)†	107 (84.3%)	47 (73.4%)	57 (90.5%)	0.013
Subjective stress during the tasks	4.50 (1.53)	4.41 (1.59)	4.60 (1.48)	0.474
Sleep problems (score)	2.85 (1.34)	1.72 (0.39)	4.00 (0.92)	< 0.001

Data are presented as means (standard deviation) or n (percentage). BMI = Body Mass Index; HbA1c = Glycated hemoglobin; SD = Standard deviation.

* n = 124.

† n = 127.

### 3.2 Cortisol measures over the day and in the laboratory

The average cortisol levels for the different parameters over the day and cortisol levels throughout the laboratory session can be found in Table 2. Of the 129 participants who took part in the study, 114 participants had complete information on daily cortisol and 117 had full information on cortisol in the laboratory. These missing data were due to assay issues as reported previously (Hackett et al., 2019). We found a significant main effect of trial for daily cortisol (*F* (2.85, 322.13) *=* 98.67*, p <* 0.001). In line with expected diurnal patterning (Adam and Kumari, 2009) values were highest 30 minutes after waking 27.30 ± 16.56, then declined gently across the day to reach the lowest point in the evening 5.70 ± 5.72. We also found a significant main effect of trial for cortisol in the laboratory (*F* (2.33, 235.85) *=* 45.07*, p <* 0.001). Cortisol concentrations dropped immediately after the stress tasks, with an average decrease of 1.17 ± 2.98 nmol/l from baseline and continued to decline across the session with average declines of 2.05 ± 3.99, 2.41 ± 9.04 and 2.75 ± 6.13 nmol/l 20, 45 and 75 minutes later.

**Table 2:** Mean cortisol values in the laboratory and over the day.

	*n*	Mean (SD)
Diurnal cortisol		
Waking cortisol (nmol/l)	123	20.13 (11.82)
Cortisol awakening response (nmol/l)	121	6.72 (16.18)
Slope across the day (nmol/l per h)	124	0.02 (0.02)
Evening cortisol (nmol/l)	122	5.70 (5.72)
Diurnal cortisol AUC (nmol/l)	114	157.83 (74.20)

**Laboratory cortisol**		
Baseline (nmol/l)	125	10.16 (5.46)
Immediately post-task (nmol/l)	123	9.01 (4.65)
20 minutes post-task (nmol/l)	121	8.00 (4.10)
45 minutes post-task (nmol/l)	122	7.72 (8.53)
75 minutes post-task (nmol/l)	122	7.30 (5.67)
Laboratory cortisol AUC (nmol/l)	117	674.39 (326.45)

AUC = Area under the curve.

### 3.3 Sleep problems and cortisol over the day

**Fig. 1:**
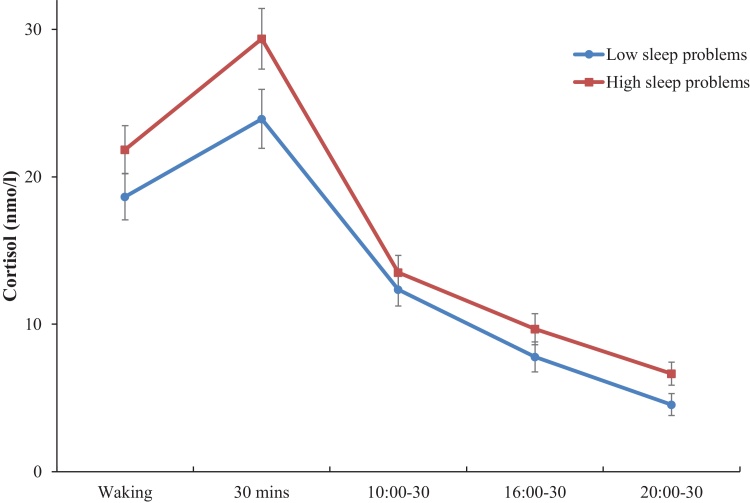
Cortisol responses for high and low sleep problem groups over the day. Values are adjusted for age, gender, education, income, marital status, body mass index and smoking status. Error bars are standard error of mean.

Sleep problems were positively associated with daily cortisol AUC (*B* = 17.051, *C.I.* = 6.547 to 27.554, *p* = 0.002) in fully adjusted models, suggesting that those with greater sleep problems had greater cortisol concentrations over the course of a normal day. Participants reporting greater sleep problems also had raised evening (20:00-20:30) cortisol levels (*B* = 0.96, *C.I.* = 0.176 to 1.746, *p* = 0.017) adjusting for age, sex, marital status, education, household income, BMI and smoking. No significant associations were found for waking cortisol levels, the slope or the CAR (*p’s* > 0.102). Fig. 1 illustrates the pattern of cortisol responses over the day in participants who reported low or high sleep problems.

### 3.4 Sleep problems and laboratory cortisol measures

**Fig. 2:**
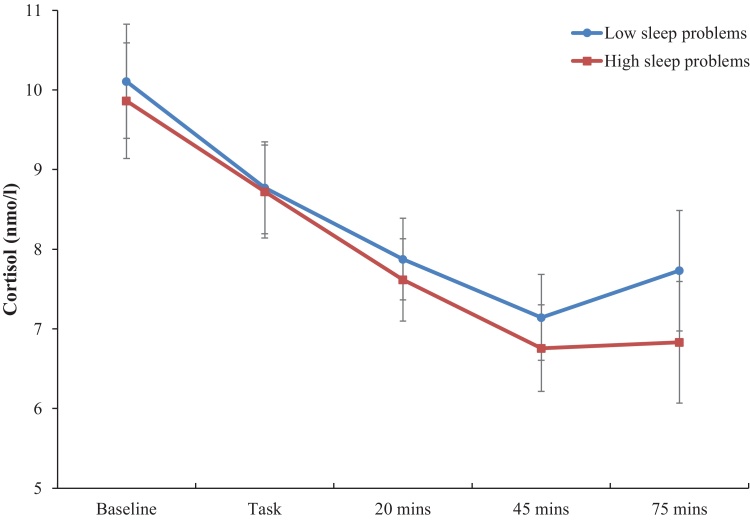
Cortisol responses for high and low sleep problem groups over the laboratory session. Values are adjusted for age, gender, education, income marital status, body mass index and smoking status. Error bars are standard error of mean.

In regression analyses, there was no association between sleep problems and baseline cortisol concentrations (*B* = -0.025, *C.I.* = -0.055 to 0.005, *p* = 0.107). However, sleep problems were negatively associated with cortisol immediately post-task adjusting for age, sex, marital status, education, household income, BMI and smoking (*B* = -0.030, *C.I.* = -0.059 to 0.000, *p* = 0.048), with a greater decrease in cortisol observed in those with reporting greater sleep problems. Sleep problems were also negatively associated with cortisol levels 45 minutes post-task in adjusted models (*B* = -0.037, *C.I.* = -0.072 to -0.002, *p* = 0.039). There was a trend towards significance for sleep problems and cortisol 20 minutes post-task (*B* = -0.028, *C.I.* = -0.059 to 0.004, *p* = 0.083) when adjusting for covariates. No significant associations between sleep problems and cortisol at 75-minute post-task or AUC were detected in adjusted models (*p’s* > 0.202). The pattern of cortisol responses in the laboratory session in participants reporting low or high sleep problems can be found in Fig. 2.

### 3.5 Sensitivity analyses

As cortisol output may differ depending on ethnicity, we assessed whether ethnicity was significantly associated with any of the cortisol measures. For cortisol over the day, ethnicity was significantly associated with AUC (*p* = 0.005), with white participants having higher AUC than non-white participants. No significant associations were detected between ethnicity and waking cortisol, the CAR, the slope or evening cortisol values (*p*’s > 0.088). We also investigated whether there was an interaction between sleep problems and ethnicity on any of the cortisol measures over the day. No significant interactions were detected for waking cortisol, the CAR, the slope or evening cortisol (*p*’s > 0.281). A significant interaction between ethnicity and sleep problems was detected for AUC (*p* = 0.005). However, the association between sleep problems and AUC remained significant when excluding non-white participants from the analysis (*B* = 17.15, *C.I.* = 5.40 to 28.89, *p* = 0.005).

For laboratory cortisol no significant associations with ethnicity were detected (*p*’s > 0.072). We also assessed whether there was an interaction between sleep problems and ethnicity on any of the laboratory cortisol measures. No significant interactions were detected for baseline cortisol, cortisol immediately post-task or AUC (*p*’s > 0.105). However, significant interactions between ethnicity and sleep problems on cortisol values at 20-, 45- and 75-minutes post-task were detected (*p*’s < 0.038). We further explored these interactions by removing non-white participants from our analyses. In these analyses the association between sleep problems and cortisol levels 45 minutes post-task remained significant (*B* = -0.048, *C.I.* = -0.086 to -0.011, *p* = 0.012). Again, no significant association between sleep problems cortisol at 75-minute post-task was detected (*B* = -0.025, *C.I.* = -0.065 to 0.016, *p* = 0.235). However, the association between sleep problems and cortisol 20 minutes post-task that approached significance in the main models, reached significance when removing non-white participants from the analysis (*B* = -0.035, *C.I.* = -0.069 to -0.002, *p* = 0.040)

## 4 Discussion

This study explored the link between sleep problems and cortisol in people with T2D. We observed a positive association between sleep problems and daily cortisol AUC, indicating that those reporting greater sleep problems had higher cortisol levels over the course of the day. Participants reporting greater sleep problems also had raised evening cortisol levels. In the stress laboratory, sleep problems were negatively associated with cortisol immediately and 45 minutes post-task, suggesting that those who experienced greater sleep problems had lower cortisol concentrations following stress. Our results were robust to adjustment for a range of covariates and did not change when ethnicity was taken into account in our sensitivity analyses.

We found that participants with higher sleep problem scores had greater daily cortisol AUC. Previous research concerning the link between sleep problems and diurnal cortisol is mixed. The use of varied sleep (Chrousos et al., 2016) and cortisol measures (Adam and Kumari, 2009) makes it difficult to draw clear comparisons with earlier work. Findings from studies which have used a similar observational design to the present study, along with salivary cortisol assessment are equivocal. One study of 4489 Danish civil servants reported a link between self-reported sleep problems over the past month and raised diurnal AUC (Hansen et al., 2012). However, another large study with an objective actigraphy-based sleep quality measure (Castro-Diehl et al., 2015) did not detect this association. While in contrast, a recent review concluded that insomnia is associated with raised cortisol output (Chrousos et al., 2016).

This previous work has been conducted in community samples (Castro-Diehl et al., 2015; Hansen et al., 2012) or in those with diagnosed insomnia (Chrousos et al., 2016), whereas participants in this study all had confirmed T2D. Neuroendocrine dysfunction is prevalent in T2D and people with the condition have higher cortisol AUC concentrations than those without the condition (Hackett, 2016; Steptoe et al., 2014). Our current findings suggest that sleep problems in T2D may be associated with further disturbances in daily cortisol output in this population.

We detected an association between sleep problems and elevated evening cortisol concentrations. Earlier observational work has produced diverging findings, with both raised (Kumari et al., 2009) and low evening cortisol levels (Hansen et al., 2012) related to subjective sleep problems in two cohorts of civil servants. Some experimental work has linked sleep deprivation and raised evening plasma cortisol concentrations (McEwen, 2006; Spiegel et al., 1999). However, other studies using different sleep restriction protocols have mostly failed to detect any association (Chrousos et al., 2016). More consistent evidence has been in observed in clinical samples, with studies finding raised evening cortisol concentrations in people with insomnia (Rodenbeck et al., 2002; Vgontzas et al., 2001).

People with T2D have been observed to have raised evening cortisol concentrations in previous studies (Hackett et al., 2014; Steptoe et al., 2014) and higher evening values are predictive of new onset T2D in initially healthy samples (Hackett et al., 2016). Raised evening cortisol concentrations increase the risk of CVD mortality (Kumari et al., 2011), the leading cause of death in people with T2D (Emerging Risk Factors Collaboration, 2011). Additionally, there is a recognized association between sleep problems and CVD (Sofi et al., 2014). Taken together, the results of the present study offer the possibility that sleep problems may exaggerate disturbances in evening cortisol output in people with T2D which may increase the likelihood of cardiovascular complications in this high-risk population. However, as our findings are cross-sectional, further research is required to confirm this assertion.

We failed to detect a link between sleep problems and waking cortisol, the CAR or the cortisol slope. The lack of cross-sectional association with waking cortisol or the CAR is in line with some (Kumari et al., 2009; Van Lenten and Doane, 2016; Zhang et al., 2011) but not all studies (Abell et al., 2016; Backhaus et al., 2004; Castro-Diehl et al., 2015; Hansen et al., 2012). The studies that detected a significant association differed from the present analysis in terms of sleep problems measurement, by using a sample of insomnia patients (Backhaus et al., 2004), having objective sleep measurement (Castro-Diehl et al., 2015) or a chronic self-reported measure of poor sleep (Abell et al., 2016). This may have contributed to the diverging findings. Evidence concerning morning cortisol and CAR in people with T2D is also mixed (Champaneri et al., 2012; Hackett et al., 2014; Steptoe et al., 2014). To our knowledge no previous study has assessed the links between sleep problems and morning cortisol or the CAR in people with T2D. Further research is required to assess the potential links between sleep and morning cortisol parameters in this population.

We did not observe a relationship between sleep problems and the cortisol slope. This is in agreement with some (Abell et al., 2016; Hansen et al., 2012; Van Lenten and Doane, 2016) but not all previous work (Castro-Diehl et al., 2015; Huang et al., 2017; Kumari et al., 2009). An analysis of the Whitehall II study found that sleep problems was associated with a flatter slope in decline in cortisol across the day (Kumari et al., 2009) and people with T2D have also been found to have a flatter cortisol slope in comparison to those without T2D in this cohort (Hackett et al., 2014). Therefore, the lack of association between sleep problems and the cortisol slope in the present study was contrary to expectation. In previous work on T2D, our effect size for evening cortisol was stronger than for the slope (Hackett et al., 2016), perhaps indicating that a large sample is needed to detect associations with the slope in samples with glucose disturbance. Indeed, earlier work linking the slope and sleep problems has benefitted from greater participant numbers than the present study. Future work with a larger sample size could clarify this issue.

We also investigated whether sleep problems would influence cortisol stress responsivity. We found that sleep problems were linked with lower cortisol values immediately and 45 minutes post-task, along with a trend towards significance for reduced cortisol values 20 minutes post-stress. Our findings are in agreement with an earlier study (Wright et al., 2007) which observed a blunted cortisol stress response in participants with poor sleep quality on an objective but not a subjective sleep measure. Conversely, other work has linked poor sleep quality with greater cortisol reactively to stress (Bassett et al., 2015; Goodin et al., 2012) or has observed no association at all (Brooks and Robles, 2009). Differences in the stress task employed could account for these mixed findings. The study which reported a blunted cortisol response employed a mirror tracing task (Wright et al., 2007), whereas the other studies used the Trier Social Stress Task (Bassett et al., 2015; Brooks and Robles, 2009). Further the null findings reported in one previous study should be interpreted with caution as this analysis was only conducted with a sub-sample of 35 men (Brooks and Robles, 2009).

Our participants were part of a larger trial comparing stress responses in people with T2D and healthy controls. In comparison to controls, those with T2D had blunted cortisol responsivity (Steptoe et al., 2014). In the context of our earlier work, it is possible that sleep problems could act to compound these blunted cortisol responses, exaggerating neuroendocrine dysfunction in individuals with T2D. Longitudinal work is required to confirm the direction of this relationship.

The mechanisms underlying the relationship between cortisol and poor sleep remain to be elucidated. One potential pathway involved is stress. The link between stress and sleep is likely to be bidirectional, as stress is known to reduce sleep quality (Åkerstedt et al., 2012) and poor sleep in turn, is hypothesized to be a stressor (McEwen, 2006). Under the theory of allostatic load, sleep problems are thought to contribute over time to wear and tear on the body, resulting in disturbance across multiple biological systems including the HPA axis (McEwen, 2006). We observed that participants with T2D had raised daily cortisol AUC, combined with blunted cortisol concentrations following stress. Long-term basal HPA over-activation may result in dysregulation of this system, manifested through blunted cortisol responses following stress (Miller et al., 2007). Lower cortisol stress responsivity maps onto allostatic load theory (McEwen, 2006), with individuals unable to mount an appropriate response to challenge.

An increasing body of work suggests that stress plays a role in the pathogenesis of T2D and that stress increases the risk of complications in this population (Hackett and Steptoe, 2017). Experimental work suggests that disturbed sleep is associated with metabolic disturbances relevant to T2D including glucose metabolism and insulin resistance (McEwen, 2006; Spiegel et al., 1999). In turn, sleep problems influence neuroendocrine function and cortisol plays a key role in many diabetes-relevant processes including hepatic gluconeogenesis which controls blood glucose levels and lipolysis which encourages the release of free fatty acids and the build-up of triglycerides in fat tissue. In addition, cortisol can directly reduce insulin sensitivity and secretion through activation of glucocorticoid receptors on the β-cells of the pancreas (Di Dalmazi et al., 2012). Under allostatic load theory it is postulated that repeated exposure to poor quality sleep, along with corresponding stress, will lead to disturbances in the neuroendocrine system, that will over time increase the risk of health problems (McEwen, 2006). Although the association between sleep problems and cortisol observed in the current study is small, if recurrent, this might represent a considerable health risk. This is of concern in a high-risk population such as people with T2D, particularly in light of the links between aberrant cortisol output and CVD mortality (Kumari et al., 2011), as well as the association between sleep problems and CVD risk (Sofi et al., 2014).

Our findings must be assessed in terms of strengths and weaknesses. Our study benefitted from the recruitment of a sample with well-characterized T2D, who were free of CVD. We were able to assess cortisol in two contexts, over the course of an ordinary day and in response to a standardized stress protocol. Our analyses were robust to adjustment for covariates including smoking and BMI which have been associated with poor sleep quality (Poole and Jackowska, 2018). None of the participants in our sample self-reported having a sleep disorder such as sleep apnea. Nevertheless, we still controlled for BMI which should partially account for potential confounding of associations by undiagnosed sleep disorders, as those with sleep apnea are more likely to be obese.

Our sample was predominately White and although we explored the role of ethnicity in our sensitivity analyses, our results may not be applicable to more diverse cohorts. We did not have information on the duration of diabetes. Participants with mood disorders were excluded from the study. As depression is common in T2D (Hackett and Steptoe, 2017) and is associated with sleep problems (Koyanagi et al., 2014; Poole and Jackowska, 2018), this reduces the generalizability of our findings. We did not have information on objective sleep quality, and there is evidence that associations between sleep problems and HPA axis function may differ depending on the sleep measure employed (Chrousos et al., 2016). Further, the measure we used to assess sleep problems has not been validated for use in people with T2D. We did not have information on sleep duration on the days before sample collection or whether participants napped on the day when they provided samples outside of the laboratory environment. The influence of sleep problems on cortisol stress responsivity could vary depending on the selected stress task. Social-evaluative tasks elicit greater cortisol responses than the tasks in the present study (Dickerson and Kemeny, 2004). It is possible that different effects may have been observed using a social-evaluative paradigm. The bidirectional interrelationships between sleep problems, cortisol and stress make it difficult to draw casual conclusions. Our study was cross-sectional, and cortisol was only assessed once in the laboratory and once over the course of the day. Therefore, causal relationships between sleep problems and neuroendocrine disturbances in people with T2D cannot be drawn. Longitudinal research would help explain the extent to which chronic sleep problems and changes in sleep over time are associated with neuroendocrine dysfunction.

Despite these considerations, this study demonstrated associations between sleep problems and cortisol over the day and in response to stress in a sample of people with T2D. These findings offer the possibility that sleep-related neuroendocrine disturbance may increase the risk of CVD in this population, though further work is required to test this assertion.

## Funding

This work was supported by the 10.13039/501100000274British Heart Foundation (Grant RG/10/005/28296). The funder had no involvement at any stage of the study.

## Declaration of Competing Interest

None.
